# Associations Between Non-Genetic Risk Factors and DNA Methylation Alterations in Barrett’s Esophagus and Its Progression to Esophageal Adenocarcinoma

**DOI:** 10.3390/ijms262311704

**Published:** 2025-12-03

**Authors:** Nastaran Riahi Dehkordi, Kristi Kruusmaa, Kausilia K. Krishnadath, Arianna Bertossi

**Affiliations:** 1 Faculty of Medicine and Health Sciences, University of Antwerp, 2000 Antwerpen, Belgium; nastaran.riahidehkordi@student.uantwerpen.be; 2 Research and Development, Universal Diagnostics d.o.o., 1000 Ljubljana, Slovenia; kristikruusmaa@gmail.com (K.K.); arianna.bertossi@universaldx.com (A.B.); 3 Laboratory of Experimental Medicine and Paediatrics, Department of Gastroenterology and Hepatology, University of Antwerp, University Hospital Antwerp, 2000 Antwerpen, Belgium

**Keywords:** esophageal adenocarcinoma, Barrett’s esophagus, epigenetics, risk factors, DNA methylation

## Abstract

Esophageal cancer (EC) is one of the most aggressive cancers of the digestive system, with two main subtypes: esophageal squamous cell carcinoma (ESCC) and esophageal adenocarcinoma (EAC). Over four decades, the frequencies of EAC and Barrett’s esophagus (BE), the known precursor lesion for EAC, have sharply increased in North America and Europe. This is mainly due to lifestyle and risk factors such as gastroesophageal reflux disease (GERD), obesity, and smoking. BE development to EAC involves numerous molecular modifications, including genetic and epigenetic alterations. Epigenetic changes, such as aberrant DNA methylation, play a critical role in the pathogenesis and progression of BE. This review discusses how non-genetic risk factors contribute to DNA methylation changes driving the transformation from BE to EAC, providing insights into the potential of developing methylation-based biomarkers for early diagnosis, risk stratification, and therapeutic intervention.

## 1. Introduction

With over 400,000 deaths annually, esophageal cancer (EC) is known as one of the most aggressive and deadliest cancers worldwide [1]. Esophageal squamous cell carcinoma (ESCC) and esophageal adenocarcinoma (EAC) are the two most common histological subtypes of EC [2,3]. Despite advances in cancer diagnosis and treatment, EC remains a poorly treatable disease due to its late diagnosis. The average 5-year survival rate of EC is around 20%, with EAC having better median survival rates than ESCC, especially when the disease is detected at early stages [2,4]. Although the most prevalent form of EC worldwide is ESCC, with the highest rates characterizing Central Asia, East, and South Africa, EAC incidence has been steadily rising over the last four decades in Western countries, with annual increases of 5% in Western Europe and up to 7–8% in the US [3,5].

The increasing incidence of EAC has been associated with a concurrent increase in the incidence of Barrett’s Esophagus (BE), the only known precancerous condition for EAC [5]. The metaplasia-dysplasia-adenocarcinoma (MDA) sequence is the predominant pathway for EAC development, even though just a small percentage of BE cases transform into cancer [6].

Current screening and monitoring techniques for BE rely mainly on endoscopic and histological examinations. However, endoscopic surveillance is invasive and not a cost-effective strategy [7,8], highlighting the need for new non-invasive diagnostic tools for risk stratification of BE and early EAC.

In recent decades, there has been an upturn in epigenetics research, representing the basis for identifying new biomarkers for the screening, early detection, diagnosis, staging, and risk stratification of different malignancies, particularly through non-invasive approaches such as liquid biopsy [9,10]. Liquid biopsy, which analyzes body fluid samples, offers advantages over traditional tissue biopsy, including easy reproducibility, convenient insights into tumor burden, and a molecular snapshot of primary tumors [11]. Despite its significant potential for early cancer screening and diagnosis, challenges remain, particularly the need for highly sensitive methods at the earliest disease stages, when tumor DNA in body fluids is minimal and the genetic profile of the primary tumor is still unclear [12]. In this context, DNA methylation has gained increasing attention in recent years as a promising epigenetic biomarker in esophageal cancer research, both in tissue and blood, due to its unique cancer-specific methylation patterns, presence in the initial stages of the disease, biological stability, and technical repeatability [11,13,14].

Although several lifestyle-related factors, such as gastroesophageal reflux disease (GERD), along with obesity and smoking, have been implicated in the development of BE and its progression to EAC [15], their precise influence on epigenetic mechanisms, particularly DNA methylation, remains incompletely understood. This review aims to provide an overview of the current literature linking some non-genetic risk factors to DNA methylation alterations during the progression of BE to EAC. We highlight how understanding these changes can be crucial in developing novel clinical and diagnostic strategies for BE/EAC patients.

## 2. Barrett’s Esophagus and Esophageal Adenocarcinoma

### 2.1. Definition and Diagnosis of BE

BE is characterized by replacing the stratified squamous epithelium in the lower part of the esophagus with columnar epithelium, which is referred to as metaplasia [16]. This condition occurs due to GERD, associated with long-term exposure to stomach acids and bile salts, which flow back into the esophagus, leading to constant inflammation and tissue damage [17].

The diagnostic criteria for BE vary between different guidelines and are based on endoscopic findings and histological confirmation of tissue biopsy samples, particularly regarding the need to detect goblet cells in biopsy samples of the esophagus histologically. In the USA, histological confirmation of intestinal metaplasia (IM) with goblet cells is required, while in the UK and Japan, BE is diagnosed with columnar metaplasia of the esophagus, even in the absence of goblet cells [18,19,20].

### 2.2. BE Classification and Progression

**Figure 1:**
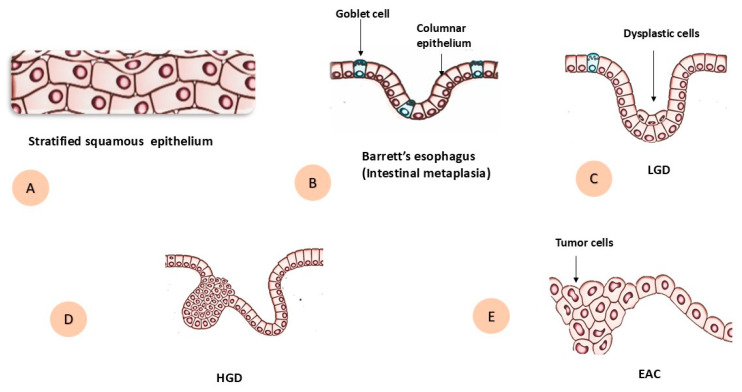
Schematic diagram during progression of Barrett’s esophagus to esophageal adenocarcinoma. (**A**) Normal stratified squamous epithelium. (**B**) Barrett’s esophagus with intestinal metaplasia, showing columnar epithelium with goblet cells. (**C**) Low-grade dysplasia (LGD) with dysplastic epithelial cells. (**D**) High-grade dysplasia (HGD). (**E**) Invasive esophageal adenocarcinoma (EAC) with tumor cells.

The progression of BE to EAC is a gradual process, and the progressive steps are histologically classified as different grades of dysplasia: non-dysplastic BE (NDBE) and dysplastic BE, which is further subclassified into low-grade dysplasia (LGD) and high-grade dysplasia (HGD) [21] (Figure 1). The annual risk of progression from NDBE to HGD or EAC is relatively low at 0.2–0.5% [22]. Whereas reported rates for LGD are considerably higher than those for NDBE, they remain highly variable across studies, mainly depending on agreement between pathologists. Due to significant inter-observer variation, LGD is frequently misclassified or underestimated in routine practice. Some studies report progression rates of <1.5% per patient-year, while others describe rates as high as 13.4% per patient-year [23]. In contrast, HGD represents the highest-risk stage, with older follow-up series reporting progression rates up to 15–20% per patient-year, although more recent European data suggest a lag time of 1.5–10 years to adenocarcinoma, corresponding to annualized risks of approximately 6–7% per patient-year [24].

Currently, the grade of dysplasia in BE is the most reliable indicator for predicting the progression towards EAC [25], and endoscopic surveillance is recommended by clinical guidelines for individuals with BE to detect dysplasia or cancer at an early stage [26,27].

### 2.3. Risk Factors for BE and EAC

#### 2.3.1. Demographics and Lifestyle Factors

The incidence of EAC and BE rises with age. The striking sex disparity in both cases is an intriguing and largely unexplained observation: the incidence rates of EAC and BE in women remain substantially lower than those in men in all countries [28]. BE and EAC are approximately twice and seven times more prevalent in men than women, suggesting that gender-related factors contribute to the formation of both BE and EAC [29].

White ethnicity is also reported as a risk factor for BE and EAC patients. EAC is more common in non-Hispanic White people compared to non-White people. Similarly, BE is less common in Hispanic and Black individuals and is rare among Asian people, while the highest rate was reported among non-Hispanic White people [30,31].

Smoking, alcohol consumption, obesity, diet, and physical inactivity are recognized as lifestyle factors that are linked to different cancers [32]. Among these factors, smoking and obesity (measured by body mass index (BMI) or waist-to-hip ratio (WHR)) are considered primary risk factors for BE and EAC [28,33]. Ever smoking, which is defined as either present or prior history of smoking, was found to be a significant predictor of increased risk for progression from NDBE and LGD-BE to HGD-BE or EAC [34].

#### 2.3.2. Anatomical and Clinical Features

BE characteristics, which are defined as baseline dysplasia status (e.g., LGD-BE vs. NDBE) and length of BE segment (per cm), are considered one of the main risk factors associated with the progression to EAC [34]. Generally, BE length is reported following the Prague C and M criteria, which depict the circumferential length (C) and the maximal length (M) of the Barrett’s epithelium’s expansion from the top of the stomach folds into the distal esophagus [35,36]. A cut-off value of 3 cm has been utilized to distinguish between patients with short-segment Barrett’s esophagus (SSBE) and long-segment Barrett’s esophagus (LSBE) [37,38]. The risk of EAC development varies with the length of the esophagus lined by Barrett’s metaplasia. While SSBE is more prevalent than LSBE, patients with LSBE have the highest risk of malignancy [39,40].

The term GERD refers to the chronic reflux of stomach acid and/or bile fluid into the esophagus, which is promoted by a hiatus hernia, obesity, and smoking, and has a crucial role in the development of BE [19]. The degree of Barrett’s metaplasia in patients is directly related to the severity of the underlying GERD. Untreated patients with LSBE often experience severe GERD and erosive esophagitis, while SSBE may not be linked to GERD symptoms or reflux esophagitis [41].

Several articles indicated an association between hiatus hernia and an increased risk of BE. A meta-analysis discovered that hiatus hernia remained a significant risk factor even after controlling for confounders like GERD [33,42,43,44]. Notably, some studies revealed that *Helicobacter pylori* infection and anemia, as well as some medications such as statins and selective serotonin reuptake inhibitors (SSRIs), acted as protective factors against the development of BE [45,46].

### 2.4. Molecular Pathogenesis of BE and EAC

#### 2.4.1. Genomic Alterations

Both BE and EAC exhibit loss of heterozygosity (LOH), aneuploidy, clonal diversity, and several genetic mutations [6]. In BE and EAC, LOH at 17p, 5q, 9p, and 13q is a frequently observed event [47,48]. The tumor suppressor genes *TP53* and *CDKN2A* are located on chromosomes 17p and 9p, respectively, and research has shown that LOH occurs frequently as a result of mutations (*TP53* and *CDKN2A*) or of promoter hypermethylation (*CDKN2A*) [6]. The 9p LOH event occurs before 17p LOH and is widespread throughout the BE lesion [49]. 17p LOH leads to genomic doubling to a 4N state, which aligns with the impact of p53 loss on genome instability. Data suggest that *CDKN2A* loss initiates BE progression, while *TP53* alterations are later events linked to neoplastic progression and aneuploidy [50]. Beyond aneuploidy, BE development has been linked to increased clonal diversity [51]. Indeed, some researchers proposed the existence of genomically distinct clones within the BE lesions, with evidence indicating that certain clones may eventually dominate over time, a process referred to as “clonal sweep” [6,52,53]. In addition, mutations can happen within specific genes, leading to loss of gene expression in tumor suppressors such as p53 and p16 [54]. In a large sequencing study by Weaver et al., 26 genes commonly mutated in EAC were analyzed in a collection of NDBE, HGD-BE, and EAC samples. The remarkable finding of this study was that there were no significant differences in mutation rates between BE and EAC samples except for two specific genes, *SMAD4* and *TP53*. Indeed, 70% of HGD-BE and EAC patients, against just 2.5% of NDBE patients, had a *TP53* mutation. In contrast, *SMAD4* mutations were identified only in EAC patients, with a frequency of 13%, highlighting a clear genetic difference between EAC and HGD [55].

#### 2.4.2. Epigenomic Alterations

Cancer exhibits various pathological characteristics, such as altered cellular differentiation, proliferation, infiltration, and metastasis. Its occurrence results from a multifaceted, intricate process strongly influenced by genetic and epigenetic factors [56]. Compared to the slower process of genomic evolution, epigenetic alterations occur more rapidly, making them more prevalent in cancer cells [9]. Epigenetics differs from genetics in that it studies changes in gene expression caused by non-genetic sequence alterations, such as DNA methylation, histone modifications, non-coding RNA regulation, chromatin remodeling, and nucleosome localization [10,50]. Over the past several decades, extensive research has investigated the role of epigenetic changes in the pathogenesis of BE and its progression to EAC, revealing that many alterations found in EAC are already present in BE, with aberrant DNA methylation being the most widely studied epigenetic mechanism in both conditions [57,58,59].

## 3. DNA Methylation in BE and EAC

Methylation control is essential for maintaining cell proliferation and metabolism, whereas abnormal DNA methylation can result in multiple diseases, including tumors. Aberrant DNA methylation affects gene expression, and, as a result, alterations of this process may contribute to oncogenesis [10]. DNA methylation is a process in which a methyl group is covalently transferred to the C-5 position of a cytosine ring of DNA by DNA methyltransferase (DNMT) enzymes. DNMTs are aberrantly expressed in various diseases, and their expression is notably elevated in many malignancies [60,61]. More than 98% of DNA methylation occurs in somatic cells within a cytosine–phosphate–guanine (CpG) dinucleotide context. In comparison, in embryonic stem cells, this percentage is reduced to 75%, with up to one-quarter of DNA methylation taking place in a non-CpG context (CHG or CHH, where H is A, T, or C) [10,62]. Generally, aberrant DNA methylation in cancer can be divided into global hypomethylation and regional hypermethylation, which occurs when methylation affects specific genomic regions such as CpG islands or gene promoters [63]. While promoting hypermethylation can cause cancer by silencing tumor suppressor genes, DNA hypomethylation is linked to genomic instability and increased expression of oncogenes [64].

In this line, many genes involved in cell cycle control, proliferation, survival, apoptosis, reactive oxygen species (ROS)-mediated DNA damage, and DNA repair have been identified as hypermethylated in BE and EAC [58,59,65]. Beyond promoter CpG island hypermethylation, the BE-dysplasia-EAC sequence exhibits extensive hypomethylation in intergenic and repetitive regions. The best example here is the hypomethylation of *LINE-1/Alu*, which is evident in BE and is frequently more pronounced in dysplasia and EAC, consistent with progressive progression [65,66,67]. Functionally, lack of methylation at repetitions causes chromosomal instability and may lead to *LINE-1* expression, retro transposition, and aberrant gene regulation [68,69].

### 3.1. Technologies for Assessing DNA Methylation

Since the first human genome was sequenced in 1990, progress in genomics, transcriptomics, proteomics, epigenomics, and microbiomics has advanced profiling of BE and its progression to EAC [70]. In the next section, we distinguish low- from high-throughput methods and highlight DNA methylation as our primary target for early detection and therapy monitoring [71].

#### 3.1.1. Targeted Locus-Specific Methylation Assays

##### MSP

Methylation-specific polymerase chain reaction (PCR; MSP), introduced by Herman in 1996, is a popular method for assessing methylation status in specific DNA regions. It involves bisulfite treatment of DNA and the use of two primers for detecting methylated and unmethylated sites. This technique is effective in identifying methylation at nearly all CpG sites in CpG islands but has limitations; it can only analyze a few CpG sites at once and provides qualitative, rather than quantitative, results regarding methylation status [72].

##### MethyLight

The MethyLight method, an advancement of MSP that overcomes its quantification limits, uses real-time fluorescence PCR to read probe signals and quantifies DNA methylation from Ct values, making it particularly effective for detecting rare low-frequency methylated DNA amid abundant unmethylated DNA [73].

##### Digital MSP

Digital PCR is a third-generation technology developed following traditional PCR and quantitative PCR. It operates as an endpoint method, eliminating the need for a standard curve for absolute quantification of nucleic acids. By dividing samples into over 20,000 droplets, it allows for random distribution across chambers, ensuring some contain one or no nucleic acid. This method significantly enhances the sensitivity and specificity for detecting DNA methylation and allows for precise absolute quantification [74].

#### 3.1.2. Array-Based Profiling

Methylation microarray technology enables comprehensive and quantitative assessment of targeted methylation sites across the genome with high-throughput capabilities, reducing costs per sample and enhancing measurement accuracy independent of read depth. The Illumina Infinium HumanMethylation450 BeadChip (450K), Infinium MethylationEPIC BeadChip (850K), and Infinium MethylationEPIC v2.0 BeadChip (935K) are prominent commercially available methylation chips, covering approx. 450,000, 850,000, and 935,000 CpG sites, respectively [75,76].

#### 3.1.3. Sequencing-Based, Genome-Wide Profiling

##### Bisulfite-Based

The conversion of genomic DNA via sodium bisulfite treatment is the leading method for distinguishing unmethylated from methylated cytosines in DNA methylation analysis. This treatment converts unmethylated cytosines to uracils, while 5-methylcytosine and 5-hydroxymethylcytosine remain unchanged [77]. Common bisulfite conversion-based sequencing techniques include whole-genome bisulfite sequencing (WGBS), reduced representation bisulfite sequencing (RRBS), and oxidative bisulfite sequencing (oxBS-seq) [78]. WGBS is the gold standard for detecting DNA methylation, providing a high-resolution single-nucleotide map of 5mC across the genome [79]. However, the harsh conditions of bisulfite treatment damage DNA, leading to fragmentation, loss of material, and pronounced GC bias in sequencing data [80].

##### Affinity Enrichment-Based

The principle of affinity enrichment-based methods involves using antibodies against 5mC or methyl-CpG binding proteins to selectively capture methylated genomic DNA fragments, exemplified by Methylated DNA ImmunoPrecipitation sequencing (MeDIP-seq) [78]. MeDIP-seq is considered a cost-effective alternative to WGBS due to lesser data requirements and lower costs; however, it has limitations, including a resolution of only 150 to 200 base pairs (bp) and bias toward hypermethylated regions due to antibody specificity [81]. Other techniques like methylation-sensitive restriction enzyme digestion sequencing (MRE-seq) also play a significant role, and its integration with MeDIP-seq can enhance genome-wide DNA methylation profiling efficiency [82].

##### Enzymatic Conversion (EM-seq)

Enzyme-based conversion, known as Enzymatic methyl-seq (EM-seq), is a method for identifying modified cytosines such as 5mC and 5hmC. EM-seq detects changed cytosines by a two-step enzymatic conversion process involving three enzymes, including Tet methylcytosine dioxygenase 2 (TET2), T4 phage β-glucosyltransferase (T4-BGT), and apolipoprotein B mRNA editing enzyme catalytic subunit 3A (APOBEC3A) [83]. Compared with harsh bisulfite conversion, EM-seq’s enzymatic workflow is gentler and causes far less DNA damage. As a result, conversion is more complete, library inserts are longer, and library yields are often higher while requiring fewer PCR cycles than WGBS. EM-seq also produces more uniform CpG coverage, better preserving the true methylation landscape of the original sample, and can recover a greater number of CpGs at lower sequencing depths, with strong consistency across different input amounts [83,84,85].

### 3.2. Clinical Implications and Biomarker Potential

In BE, biomarkers can be classified as follows: (1) histological characteristics of biopsy specimens; (2) genomic changes; (3) epigenetic markers; and (4) protein expression [86]. During the transition from IM to LGD, HGD, and EAC cells progressively acquire characteristics such as resistance to apoptosis, uncontrolled proliferation, angiogenesis, and metastasizing potential. These cellular transformations are accompanied by structural alterations in tissue architecture, increasing genomic instability, tumor-supportive microenvironment emergence, and immune response changes. As a result, these pathological processes can be detected in tissue samples or in body fluids (such as serum, plasma, mucus, or urine), manifesting as distinct genomic, epigenomic, proteomic, or metabolomic profiles. Therefore, biomarkers may originate from any of these sources and serve as indicators of pathological and physiological changes [87,88].

#### 3.2.1. Methylation Profiling in Esophageal Exfoliated Cells

Endoscopic tissue biopsies are commonly used to detect abnormal methylation in patients with EC. However, non-invasive technologies like the EsophaCap, Cytosponge and EsoCheck are now being utilized to collect cells from the esophageal mucosa [63,89]. These approaches have included ingestible cell-collection devices paired with biomarkers. The BE biomarkers assessed in these studies include protein markers (e.g., trefoil factor 3), methylated DNA markers (MDMs), and microRNAs [90,91].

In a prospective validation study of utilizing EsophaCap, a four-gene methylation panel (*p16/CDKN2A*, *NELL1*, *AKAP12*, *TAC1*) analyzed with the methylation-on-beads method discriminated BE from controls with high accuracy (sensitivity 78.6%; specificity 92.8%). This data supports EsophaCap as a safe, low-cost, non-endoscopic sampling device for methylation-based BE screening [91]. In a study conducted by Chettouh et al., the Cytosponge was used to collect esophageal cells, and DNA methylation of four markers (*TFPI2*, *TWIST1*, *ZNF345*, and *ZNF569*) was quantified by MethyLight PCR. These markers distinguished BE from reflux controls; *TFPI2* alone yielded ~79% sensitivity at ~97% specificity, while *ZNF345* achieved 100% specificity. Performance improved with increasing BE segment length, supporting Cytosponge-plus-methylation as a practical, non-endoscopic screening approach [92]. Using the swallowable EsoCheck balloon to sample esophageal cells, the study applied a two-gene methylated DNA assay (EsoGuard) that quantifies *VIM* and *CCNA1* methylation by next-generation sequencing in a high-risk BE population. In the primary analysis set, EsoGuard/EsoCheck detected BE with 87.5% sensitivity and 81.2% specificity [93]. Notably, EsoGuard uses bisulfite-based next-generation sequencing (NGS) for detection, whereas most assays for DNA methylation in esophageal exfoliated cells rely on quantitative methylation-specific PCR (qMSP) [94].

#### 3.2.2. Methylation Profiling in Circulating Cell-Free DNA

Blood testing is a minimally invasive approach, and DNA methylation can be profiled on blood specimens using standard high-throughput laboratory methods, which are often more cost-effective than analyses of esophageal exfoliated cells [94,95]. Such assays function both as cancer screening tools and as safer, more practical means to monitor treatment response in clinical practice [96]. Plasma is generally preferred to serum given the lower circulating tumor DNA (ctDNA) proportion, greater background signal, and longer fragment lengths observed in serum [97,98]. The input volume of plasma or serum strongly influences circulating cell-free DNA (cfDNA) recovery and downstream methylation performance. Insufficient plasma volume reduces cfDNA yield and degrades sample quality, thereby diminishing both the analytical sensitivity and specificity of methylation assays. To improve detection sensitivity in clinical settings, larger blood draws are commonly used; nevertheless, excessive collection can cause participant discomfort and reduce compliance. Accordingly, contemporary cfDNA methylation protocols typically recommend collecting ~10 mL of whole blood, which yields ~3–4 mL of plasma for analysis [94,99].

A further limitation of cfDNA assays is reduced specificity from extra-tumoral signal, whereby fragments shed by noncancerous tissues or other malignancies may generate false positives. In addition, blood-based DNA methylation markers have relatively low sensitivity in early-stage disease, irrespective of the detection platform. The lower sensitivity in blood is largely due to the reduced abundance of ctDNA compared with tissue. Together, these drawbacks diminish their clinical applicability [94,100,101].

In a study by Jin et al., *TAC1* methylation was assessed in paired plasma and tissue for EAC: specificity was similar (91.4% vs. 92.5%), but sensitivity was markedly lower (29.5% vs. 61.2%) [102]. You et al. reported a similar pattern for ESCC patients: methylated *PTPRO* showed 75.0% sensitivity in tissue but only 36.1% in plasma [103]. However, a panel of markers is likely the better strategy for methylation screening in blood. Qin et al. identified 23 tissue-derived methylation candidates with sensitivity for both EAC and ESCC, then shortlisted 12 markers for plasma evaluation. They narrowed the list to five markers (*FER1L4*, *ZNF671*, *ST8SIA1*, *TBX15*, and *ARHGEF4*), creating a panel that detected EAC and ESCC with 74% and 78% sensitivity, respectively, and 91% specificity [104].

### 3.3. Non-Genetic Risk Factors and DNA Methylation Alterations

Ongoing research in BE and EAC is increasingly focused on identifying hyper-methylated genes with diagnostic or prognostic value, as well as understanding their correlations with non-genetic variables such as lifestyle and environmental exposures [90,92,105,106]. Notably, only a few studies have specifically examined how DNA methylation changes relate to modifiable risk factors in the context of BE and EAC. Subsequently, no methylation-based biomarker has yet been adopted in routine clinical practice for BE surveillance or EAC management due to insufficient validation in clinical trials [92]. In this review, we summarize current knowledge on aberrant DNA methylation in selected genes and examine their links to non-genetic influences, highlighting their potential roles in disease progression and their emerging value as diagnostic biomarkers.

#### 3.3.1. Obesity-Related Methylation Changes

Obesity is characterized by chronic low-grade inflammation driven by adipose-derived cytokines/adipokines (e.g., interleukin-6 (IL-6), plasminogen activator inhibitor-1 (PAI-1), and C-reactive protein (CRP)) and immune remodeling in adipose tissue [107,108]. These signals recruit and polarize macrophages and neutrophils, activating nuclear factor kappa B (NF-κB)/Janus kinase/signal transducer and activator of transcription (JAK/STAT) pathways and the NLR family pyrin domain containing 3 (NLRP3) inflammasome, which elevates IL-1β and IL-18 and promotes a tumor-supportive microenvironment in the upper gastrointestinal (GI) tract [109]. Neutrophil extracellular traps (NETs) can further stimulate Toll-like receptor 4 (TLR4)-dependent epithelial signaling and cytokine induction (IL-1β, IL-6, IL-8) [110]. Chronic adipokine/innate-immune signaling activates NF-κB/STAT3, which is linked to epigenetic reprogramming by increasing DNMT activity and perturbing TET enzymes, offering a mechanistic route to locus-specific hypermethylation at inflammatory genes [108,111]. 

While multiple previous studies have linked elevated BMI to altered DNA methylation [112,113,114], so far, few studies have examined the relationship between obesity, epigenetic alterations, and BE/EAC pathogenesis [115]. Obesity may contribute to the development of three esophageal disorders: GERD, BE, and EAC [116]. Recent research indicates that while obesity was traditionally believed to exacerbate GERD by increasing abdominal pressure, visceral adipose tissue (VAT) also contributes independently to esophageal inflammation. This occurs through the production of pro-inflammatory cytokines that disrupt the gastroesophageal mucosa. Additionally, elevated leptin and reduced adiponectin levels, both secreted by visceral fat, are identified as independent risk factors for the progression of EAC [117,118].

According to emerging evidence, genes involved in pathways implicated in obesity-related malignancies and adipose-mediated inflammation (e.g., *INS*, *IGF1*) exhibit altered methylation in obese individuals [118]. In a comprehensive study conducted by Kaz et al., the methylation status of genes in obesity-related pathways and their correlation with BMI were assessed by using 450K array in esophageal tissue samples (BE, LGD, HGD, EAC) stratified by high BMI (BMI > 30 = obese) or low BMI (BMI ≤ 30). They found a total of 974 differentially methylated loci (DML) between the high and low BMI cases. Overall, high BMI cases had higher methylation levels at the DML, with 872 out of 974 (89.5%) showing elevated methylation compared to low BMI cases. Moreover, it was reported that Insulin-Like Growth Factor Binding Protein 1 (*IGFBP1*) and Insulin Receptor Substrate 2 (*IRS2*) genes, members of the insulin/IGF-1 pathway, were hypermethylated in the high-BMI BE patients compared to the low-BMI cases. Furthermore, it was observed that HGD-BE and EAC lesions from patients with high BMI are characterized by hypermethylation of the proinflammatory gene Interleukin 1 Beta (*IL1B*) [119]. To identify biological processes or pathways that were over- or under-represented among genes with DML in esophageal tissue samples from subjects with high or low BMI status, Kyoto Encyclopedia of Genes and Genomes (KEGG) and the list of Gene Ontology (GO) terms were utilized. Based on the results, one KEGG pathway (“Wnt signaling”) and 87 GO terms (including “tissue morphogenesis” and “response to TGF-beta”) showed differential methylation between HGD/EAC cases in individuals with high BMI compared to those with low BMI [119].

#### 3.3.2. GERD-Related Methylation Changes

GERD and BE are associated with an inflammatory condition [120], and this status not only promotes direct DNA damage and genetic alterations but also influences DNA expression through epigenetic mechanisms, including altered DNA methylation [121]. Cellular oxidative stress, caused by an imbalance between ROS production and antioxidant responses, plays an essential role in cancer development [122]. It is well established that inflammation is a key trigger of ROS production. High levels of ROS are harmful to the cells and can cause damage to nucleic acids, proteins, lipids, membranes, and organelles [123,124]. In GERD, recurrent reflux of gastric juice and bile salts induces H_2_O_2_ production and elevates intracellular ROS levels, leading to oxidative DNA damage and double-strand breaks [125].

Glutathione S-transferase (GST) and glutathione peroxidase (GPX) enzymes are key antioxidants protecting cells from ROS damage. Peng et al. found that chronic inflammation in GERD may epigenetically inactivate these defenses. In EAC cases, they observed promoter hypermethylation of *GPX3*, *GPX7*, *GSTM2*, and *GSTM3*; hypermethylation was likewise present in BE and Barrett’s dysplasia (BD), suggesting an early event in adenocarcinoma development, as measured by quantitative bisulfite pyrosequencing [126]. In another study, the role of *GPX3* promoter hypermethylation has been analyzed using bisulfite conversion followed by MSP in Barrett’s tumorigenesis. *GPX3* mRNA expression has been shown to be persistently reduced in EACs relative to the normal mucosa. At the same time, *GPX3* promoter hypermethylation has been identified in a substantial proportion of Barrett’s samples: 61.9% in metaplasia, 81.8% in dysplasia, and 88.2% in EAC [127]. This study suggests that *GPX3* promoter hypermethylation represents a primary mechanism of *GPX3* gene inactivation and plays a crucial role in developing BE and its carcinogenesis cascade.

Beyond the inactivation of antioxidant defenses, ROS-driven epigenetic changes also target key tumor suppressors involved in cell cycle regulation, particularly *CDKN2A* (p16). The *CDKN2A* gene inhibits cyclin-dependent kinases and is a tumor suppressor implicated in various malignancies [128]. Epigenetic alterations of *CDKN2A* represent key events in the progression of BE toward EAC, providing valuable insights for designing novel therapeutic approaches and enhancing patient risk stratification. In this context, Jie Hong et al. demonstrated that exogenous H_2_O_2_ significantly elevated *CDKN2A* promoter methylation, supporting the notion that ROS mediate acid-induced hypermethylation of *CDKN2A* and promote cellular proliferation [111]. Consistent with these findings, Chueca et al., in a more recent investigation, assessed the methylation status of 77 esophageal biopsies (BE and/or EAC), using laser-capture microdissection followed by sodium bisulfite treatment and quantitative pyrosequencing; *CDKN2A* methylation increased with lesion progression [129]. Likewise, Wang et al. demonstrated that BE patients who advanced from baseline pathology to HGD or EAC exhibited a markedly higher prevalence of *CDKN2A* hypermethylation in their initial biopsies compared with non-progressors as measured by sodium bisulfite conversion followed by MSP [130]. Moreover, Schulmann et al., using qMSP, reported that hypermethylation of *CDKN2A* occurs at early stages of BE-associated neoplastic transformation and functions as a predictive marker of disease progression [131].

Death-associated protein kinase (*DAPK*) is a tumor-suppression gene whose function is tightly associated with the p53-dependent pathway of apoptosis [132,133]. *DAPK* triggers programmed cell death, and its epigenetic silencing, mediated by promoter hypermethylation, prevents cells from undergoing programmed apoptosis [128,134]. A study performed by Kuester et al., using sodium bisulfite conversion followed by MSP, revealed that the severity of reflux esophagitis is linked to the hypermethylation of the *DAPK* gene and that changes in DAPK protein expression are likely caused by long-term inflammation in reflux esophagitis. Results from this study indicated that CpG island hypermethylation of the *DAPK* promoter happens in early-stage lesions, and it has been detected in 50% of Barrett’s metaplastic samples, in 53% of BD samples, and in 60% of EAC samples. Moreover, the concomitant reduction in protein expression has been demonstrated throughout the multistep Barrett’s carcinogenesis process. The direct correlation between the progressive rise in *DAPK* hypermethylation, the decline in protein expression, and the advancement of the neoplastic process forms the basis for the hypothesis that the inhibition of the methylation process at any stage of Barrett’s carcinogenesis might be an effective strategy to prevent cancer onset and disease advancement [135].

The mismatch repair (MMR) system preserves genomic stability by correcting replication errors [136]. Although genetic mutations in MMR genes are rare, epigenetic silencing, particularly *MLH1* promoter hypermethylation, is a common mechanism of MMR inactivation in various cancers [137,138,139]. *MLH1*, part of the MutL heterodimer, repairs single-base mismatches and insertion/deletion loops [140]. In esophageal diseases, Vasavi et al. found *MLH1* promoter hypermethylation in 88.8% of GERD patients, compared to 63.5% in EAC and 53.8% in BE, as determined by a restriction enzyme–based methylation assay. These findings suggest that reflux-induced inflammation fosters a microenvironment conducive to aberrant DNA methylation, contributing to MMR deficiency [141]. However, not all studies have reported *MLH1* promoter methylation as a frequent event in BE or EAC. In a recent targeted profiling study using qMSP, Pinto et al. found that *MLH1* promoter hypermethylation was rare in both BE and EAC, in contrast to the frequent methylation of *APC*, *CDKN2A*, *MGMT,* and *TIMP3* [142]. In line with this, Eads et al. used MethyLight to profile a panel of genes across the BE–dysplasia–EAC sequence and classified *MLH1* among loci that were only infrequently methylated, without a significant increase across histologic stages [58].

#### 3.3.3. Smoking-Related Methylation Changes

Lifestyle risk factors like smoking have long-lasting effects on the genetic and epigenetic status of the human genome, and such effects include alterations in the DNA methylation status of multiple cancer-related genes [143]. While most studies on smoking-associated methylation changes have focused on ESCC [144,145,146], little is known regarding the link between smoking and alteration of DNA methylation in BE and EAC.

To address this, Kaz et al. investigated the link between smoking and DNA methylation alterations using 450K array in esophageal tissue samples, including samples from BE and EAC patients. All patients were stratified into “smokers” (which included both current and former smokers) and “nonsmokers.” The study identified 256 DML between smokers and nonsmokers in esophageal tissues; 95% of these DML showed increased methylation in the smoker group, and 41% affected cancer-associated genes [119]. Moreover, by comparing smokers to nonsmokers among BE patients, it was reported that the *TNXB* gene, which regulates cell-extracellular matrix interactions and is a member of the tenascin family [147], was located within a differentially methylated region (DMR), indicating that its methylation status significantly differs between the two groups [119]. In other words, the *TNXB* gene presents a distinct methylation profile in smokers compared to nonsmokers, suggesting a possible association between epigenetic modifications and cigarette consumption in BE [119]. In addition, this study evaluated the methylation patterns in smokers versus nonsmokers among the HGD/EAC group. It was reported that the *GFI1* gene, a transcriptional repressor involved in controlling the activity of p53 and Notch signaling [148,149], as well as the *CLDN11* gene, which is a cell adhesion protein that is frequently altered in cancer and is implicated in cell migration, are differentially methylated in smokers vs. nonsmokers cases. Both genes are detected within DMRs [119].

Previous studies have reported hypermethylation of *NTRK2* and *NTRK3* in colon malignancies [150,151]. Polycyclic aromatic hydrocarbons and nitrosamines in tobacco smoke activate aryl hydrocarbon receptor (AhR) and maintain chronic NF-κB/STAT3 signaling, which increases DNMT1 and DNMT3A activity. At the same time, oxidative stress inhibits TET enzymes, and these combined effects favor hypermethylation of promoter CpG islands [152,153,154,155]. In this context, the smoker-biased DML reported by Kaz et al. at *TNXB* (extracellular matrix (ECM)/epithelial–mesenchymal transition (EMT)linkage), *GFI1* (p53/Notch repressor), *NTRK2/3* (neurotrophin receptors), and *CLDN11* (tight junctions) is mechanistically consistent with smoke-induced epithelial remodeling and selective silencing of differentiation/tumor-suppressive pathways [119,147,148,149,151,156,157].

Kaz et al. identified the hypermethylation of *NTRK2/3* in smokers’ HGD/EAC tissues. The differentially methylated *NTRK2* locus in a promoter CpG island revealed an average methylation level of 36% in smokers’ HGD/EAC samples, compared to 9% in nonsmokers. In contrast, the differentially methylated *NTRK3* locus in the gene body displayed a median methylation of 85% in smokers’ HGD/EAC samples compared to 62% in nonsmokers [119].

#### 3.3.4. Segment Length-Related Methylation Changes

The length of BE is a critical factor in HGD-BE/EAC development. A longer BE segment is linked to a higher risk of disease progression towards EAC [158], and aberrant methylation of several genes, such as *NELL1*, *CDH13*, *SST*, and *AKAP*12, has also been associated with BE’s segment length. These genes will be discussed in the following sections.

The *NELL1* gene is located on chromosome 11p15, a locus frequently exhibiting LOH in human malignancies, including EAC [159,160]. A study from Jin et al. demonstrated that *NELL1* promoter methylation increases early during neoplastic progression, ranging from 0% in frequency in normal esophageal tissues to 42.1% in LGD-BE, 61.9% in HGD-BE, and 47.8% in EAC. Moreover, *NELL1* hypermethylation, quantified using bisulfite-treated DNA and qMSP, was strongly correlated with Barrett’s segment length. LSBE patients were more frequently characterized by the hypermethylated *NELL1* gene than SSBE patients. In addition, *NELL1* promoter methylation status has been correlated with overall survival in patients with stage I-II EAC, suggesting its potential use as a biomarker of poor prognosis in early-stage EAC [57,161].

*CDH13* (also known as H-cadherin and T-cadherin) is a member of the cadherin gene class mapped to 16q24, acts as a tumor suppressor gene, and its downregulation has been reported in many cancer types, while being associated with poor prognosis [162]. *CDH13* hypermethylation has been detected early during the neoplastic progression of BE to EAC, ranging from 0% in normal esophagus (NE) and rising to 58.3% in BE, 77.5% in BD, and 76.1% in EAC, while being rare in ESCC, and suggesting its utility as a specific biomarker for EAC. Notably, *CDH13* methylation, quantified using bisulfite conversion and qMSP, correlated robustly with BE segment length, and hypermethylation was significantly more frequent in LSBE than SSBE. Likewise, specimens with hypermethylated *CDH13* promoters had considerably longer BE segments than the unmethylated ones, indicating that the methylation status of *CDH13* may also serve as a molecular indicator of the BE segment length [163].

Somatostatin (SST) is a growth hormone-inhibitory peptide expressed in both endocrine and non-endocrine tissues, including the gastrointestinal tract, where it regulates motility, secretion, and absorption, and most notably acts as a potent inhibitor of gastric secretion. In addition to its physiological roles, SST also suppresses tumor cell proliferation, and its promoter hypermethylation has been reported in colon cancer [164,165]. Considering these findings, it has been hypothesized that the *SST* gene may also be epigenetically silenced in esophageal cancers. Jin et al. analyzed 260 esophageal tissue samples, using bisulfite conversion and qMSP. Their results showed a marked increase in *SST* promoter hypermethylation from 9% in NE tissues to over 70% in BE, HGD, and EAC samples, with intermediate levels observed in LGD (63.2%) and lower levels in ESCC (53.8%). Importantly, *SST* hypermethylation was significantly more frequent in LSBE compared to SSBE, suggesting its potential as a molecular marker of segment length and a predictive biomarker for BE progression [166].

The A-Kinase Anchoring Proteins (AKAPs) are key regulators of intracellular signaling pathways. Among them, AKAP12 functions as a versatile scaffolding protein that contributes to the regulation of signal transduction, cytoskeletal organization, and tumor suppression. Its loss has been associated with enhanced cell proliferation, invasion, and angiogenesis in several types of cancer [167]. *AKAP12* hypermethylation has been reported in a variety of human cancers, including gastric and colorectal cancers [168]. Jin et al. analyzed *AKAP12* promoter methylation in 259 esophageal tissue samples and found it to be frequent in EAC but not in ESCCs as quantified by bisulfite conversion followed by qMSP. Methylation was absent in normal tissues but increased to 38.9% in BE, 52.5% in dysplastic BE, and 52.2% in EAC, indicating that *AKAP12* hypermethylation is an early and progressive event in EAC development. Moreover, it was significantly associated with longer BE segments, suggesting a link between methylation status and segment length. These findings support the potential of *AKAP12* promoter hypermethylation as a biomarker for early detection of Barrett’s-associated esophageal neoplasia [169]. An overview of the methylated genes discussed is provided in Table 1.

## 4. Conclusions

The precise cellular and molecular mechanisms contributing to Barrett’s malignant transformation/neoplastic progression to EAC have not been completely understood to date. In this matter, epigenetic alterations, mainly aberrant DNA methylation, through regulating various genes and signaling pathways, might play a significant role in the multistep process that leads to EAC development from BE lesions.

This review article emphasizes the methylation alterations that occur during the MDA sequence and that are associated with well-known non-genetic risk factors for BE and EAC, such as obesity, GERD, smoking, and BE’s segment length. Despite our knowledge of the molecular alterations that affect crucial oncogenic pathways in BE and EAC, identifying BE patients who will eventually progress to EAC remains challenging. In this review, we underscored the methylation status of several potential genes that may be useful as molecular biomarkers for early diagnosis and risk stratification in BE/EAC patients. However, the main limitation is a lack of research that directly links non-genetic risk factors to methylation changes in BE and EAC using blood or other non-invasive methods. While similar correlations have been identified in other cancers, data for BE/EAC are scarce. Addressing this gap is crucial because it highlights the need for more study into how modifiable exposures influence the epigenetic changes detected in liquid biopsies.

To fully realize the clinical potential of DNA methylation markers, future research needs to validate candidate genes in large longitudinal cohorts while investigating how non-genetic risk factors alter methylation landscapes in BE and EAC. Integrating such data with clinical risk models and non-invasive screening approaches can dramatically improve early identification, prevention strategies, and patient outcomes.

## Figures and Tables

**Table 1:** Methylated genes involved in the pathogenesis of Barrett’s esophagus and/or esophageal adenocarcinoma.

Classification	Gene	Stage(s) with Signals	Specimen	Methylation Assay	Readout	Notes	Ref(s)
Obesity-related genes	*IGFBP1*	BE (high-BMI)	Biopsy	450K/EPIC array	Quant (β-value)	Hypermethylated in BE among high-BMI	[119]
	*IRS2*	BE (high-BMI)	Biopsy	450K/EPIC array	Quant (β-value)	Hypermethylated in BE among high-BMI	[119]
	*IL1B*	HGD/EAC (high-BMI)	Biopsy	450K/EPIC array	Quant (β-value)	Hypermethylated in HGD/EAC among high-BMI	[119]
GERD-related genes	*GPX3*	BE → HGD/EAC	Biopsy	Pyrosequencing/MSP	Quant/Qual	Hypermethylation linked to oxidative stress	[126,127]
	*GPX7*	BE → HGD/EAC	Biopsy	Pyrosequencing	Quantitative	Hypermethylation linked to oxidative stress	[126]
	*GSTM2*	BE → HGD/EAC	Biopsy	Pyrosequencing	Quantitative	Detox pathway; GERD-linked	[126]
	*GSTM3*	BE → HGD/EAC	Biopsy	Pyrosequencing	Quantitative	Detox pathway; GERD-linked	[126]
	*CDKN2A*	BE → HGD/EAC	Biopsy	pyrosequencing MSP/qMSP	Qual/Quant	Hypermethylation observed in GERD-associated context across BE progression	[129,130,131]
	*DAPK*	BE → HGD/EAC	Biopsy	MSP	Qualitative	Hypermethylation reported across BE progression	[135]
	*MLH1*	Occasional in BE/HGD/EAC	Biopsy	Restriction enzyme–based	Qualitative	Less frequent than classic BE markers; GERD-associated note	[141]
Smoking-related genes	*TNXB*	BE (smokers vs. non-smokers)	Biopsy	450K/EPIC array	Quant (β-value)	Differential methylation in smokers	[119]
	*GFI1*	HGD/EAC (smokers vs. non-smokers)	Biopsy	450K/EPIC array	Quant (β-value)	Differential methylation in smokers	[119]
	*CLDN11*	HGD/EAC (smokers vs. non-smokers)	Biopsy	450K/EPIC array	Quant (β-value)	Differential methylation in smokers	[119]
	*NTRK2*	HGD/EAC (smokers)	Biopsy	450K/EPIC array	Quant (β-value)	Hypermethylated in smokers	[119]
	*NTRK3*	HGD/EAC (smokers)	Biopsy	450K/EPIC array	Quant (β-value)	Hypermethylated in smokers	[119]
Segment length -related genes	*NELL1*	NDBE → HGD/EAC	Biopsy	qMSP	Quantitative	Methylation increases with segment length; early event	[57,161]
	*CDH13*	BE, BD, EAC	Biopsy	qMSP	Quantitative	Segment-length association, risk stratification	[163]
	*SST*	NDBE → HGD/EAC	Biopsy	qMSP	Quantitative	Early/frequent hypermethylation; stronger in LSBE	[166]
	*AKAP12*	NDBE → HGD/EAC	Biopsy	qMSP	Quantitative	Early methylation; associated with longer BE segment	[169]

Abbreviations: BE, Barrett’s esophagus; NDBE, non-dysplastic BE; BD, Barrett’s dysplasia; HGD, high-grade dysplasia; EAC, esophageal adenocarcinoma; 450K/EPIC array, HumanMethylation450/Methylation EPIC BeadChip; BMI, body mass index; MSP, methylation-specific PCR; GERD, gastroesophageal reflux disease; qMSP, quantitative methylation-specific PCR; LSBE, long-segment BE.

## Data Availability

No new data were created or analyzed in this study. Data sharing is not applicable to this article.
